# Dealing with workplace violence in emergency primary health care: a focus group study

**DOI:** 10.1186/s12875-015-0276-z

**Published:** 2015-05-01

**Authors:** Tone Morken, Ingrid H Johansen, Kjersti Alsaker

**Affiliations:** National Centre for Emergency Primary Health Care, Uni Research Health, Bergen, Norway; Department of Global Public Health and Primary Care, University of Bergen, Bergen, Norway; Faculty of Public Health and Social Sciences, Bergen University College, Bergen, Norway

**Keywords:** Focus groups, Qualitative research, Workplace violence, Primary health care, Emergency care

## Abstract

**Background:**

Prevention and management of workplace violence among health workers has been described in different health care settings. However, little is known about which phenomena the emergency primary health care (EPC) organization should attend to in their strategies for preventing and managing it. In the current study, we therefore explored how EPC personnel have dealt with threats and violence from visitors or patients, focusing on how organizational factors affected the incidents.

**Methods:**

A focus group study was performed with a sample of 37 nurses and physicians aged 25–69 years. Eight focus group interviews were conducted, and the participants were invited to talk about their experiences of violence in EPC. Analysis was conducted by systematic text condensation, searching for themes describing the participants’ experiences.

**Results:**

Four main themes emerged for anticipating or dealing with incidents of threats or violence within the system: (1) minimizing the risk of working alone, (2) being prepared, (3) resolving the mismatch between patient expectations and the service offered, and (4) supportive manager response.

**Conclusion:**

Our study shows a potential for development of better organizational strategies for protecting EPC personnel who are at risk from workplace violence.

## Background

Working in emergency primary health care (EPC) is associated with a high risk of experiencing violence from patients and visitors. According to a Norwegian study, one in three EPC workers has suffered physical abuse during their career [1]. Other studies have shown that emergency departments and emergency physicians are at great risk of violence [2,3]. Violence against health personnel has also been reported as a problem in countries like the UK [4], Ireland [5], Spain [6], Australia [7], Canada [8] and the United States [9]. In a recent study of general practitioner (GP) experiences in Norwegian EPC centres, informants spontaneously expressed apprehension about personal safety [10]. Similar findings have been reported from other countries [11,12], and this apprehension may have consequences for the perception of personal safety, the efficiency of patient care [11] and participation in out-of-hours care [13].

Measures for preventing and managing workplace violence among health workers have been described in different health care settings [11,14]. Several countries have published general safety and security recommendations for health personnel [15-22]. However, the nature of workplace violence differs between parts of the health sector as well as between cultures and countries [23,24]. In Norway, the Work Environment Act states that employees should be protected as far as possible against violence and threats (§ 4–3) [25]. The availability of safety measures in EPC in Norway has previously been described [26], but there is little knowledge about the actual benefit of these measures. The lack of research on strategies which might reduce or prevent workplace violence against EPC workers has also been pointed out elsewhere [27].

Although threats and violence happen regularly at EPC centers, we have limited knowledge of how they are dealt with. Previous studies have mostly focused on the prevalence and predictors of work place violence, often on individual factors involving the health care worker, the perpetrator or the situation. Several studies have underscored the value of examining violence in a systemic perspective rather than as a strictly interpersonal conflict problem [28-30]. A systemic perspective promotes a better understanding of the complexity of the problem and the impact of contextual factors. This perspective could therefore help in identifying relevant phenomena for the management of violence. According to Cutcliffe, phenomena which contribute to aggression and violence can be grouped into four categories: environmentally-related, client-related, system-related, and clinician-related [30]. A better understanding of how personnel deal with violent incidents may point to factors the EPC organization should pay attention to in preventing and managing workplace violence. In the current study, we have therefore explored how personnel dealt with threats and violence from visitors or patients, focusing on how organizational factors affected the incidents.

## Methods

Due to the exploratory purpose of the study, we chose a qualitative design and performed a focus group study, to reduce the impact of the interviewer, as well as to encourage sharing and comparing of experiences and views among informants.

### The setting: Norwegian EPC

The focus group study was performed among health care personnel with work experience from EPC. Norwegian EPC centres are commissioned by law, and provide EPC during evenings, nights, weekends and public holidays. They are gatekeepers to secondary care. Depending on the size of the community served, the number of staff on duty at any given time varies from one to several persons, including physicians (mandatory), nurses and other health personnel. The physicians primarily see patients at the centre, but they also conduct home visits and participate on site in emergencies outside hospitals. When nurses or other health personnel are present, they perform triage in the patient’s initial contact with the centre, give advice when appropriate and assist the physician when needed.

### Participants

Participants were recruited by announcement at conferences, in a school for further education of nurses specializing in EPC, and by e-mails distributed to employees via managers at EPC centres in different parts of Norway. Some participants were also recruited through other participants. Initially the main criterion for inclusion was personal experience of threats or violence, and the potential participants were invited to contact the researchers directly by phone or by email. However, this strategy mostly recruited nurses. To access experiences of GPs, an open invitation was sent to pre-established groups to discuss the theme with one or two researchers present. The GP groups were not given any inclusion criteria apart from willingness to discuss the issue.

A total of 37 physicians and nurses were included in the study (Table 1). There was a slight majority of physicians and females. Mean age was 41 years (range 25–69). Mean length of work experience in EPC was 9 years (range 1–33). The participants had work experience from an organizationally and geographically diverse subset of Norwegian EPC centres.

**Table 1:** **Sample distribution of participants in the eight groups (n = 37)**

		**n**	**%**
Occupation	Nurse	15	41
	Physician	22	59
Gender	Female	23	62
	Male	14	38
Age	<30 years	3	8
	30-39 years	18	49
	40-49 years	8	22
	50-59 years	6	16
	>60 years	2	5
Years in emergency primary health care			
	≤5 years	18	49
	6-10 years	8	21
	>10 years	11	30

### Data gathering

Eight focus groups were convened in the period between October 2012 and November 2013. Each group comprised two to six participants. All the participants in a given group had the same profession, and three of the groups consisted of pre-established supervision groups. Before the focus group discussion started, all participants gave a written informed consent to the secretary (KA or TM) of the focus group. The participants were also asked to complete a brief form, including questions on age, occupational title and years of work experience in EPC.

All discussions were recorded by digital sound-recorder. The discussions lasted approximately 90 minutes, and each group had one meeting. The moderator (IHJ or TM) initiated the discussions by inviting everyone to talk about personal experiences of threats or violence. The discussion was structured around the questions, “Can you describe one episode of experiencing threats or violence at work in the EPC?” and “How did you manage the situation?” The group members were encouraged to talk freely.

### Analysis

Each interview was transcribed verbatim by TM or IHJ. The transcripts were audited by the co-researcher (IHJ or TM) for reliability and imported into a qualitative software package (Nvivo 10) to aid data analysis. The analysis process was based on systematic text condensation [31]. First, the transcription was read by all authors to obtain an overall impression. Emerging themes were discussed, and the further analysis focused on themes related to available strategies and organizational conditions which influenced the participants’ management of the threatening situations. Based on the selected themes, meaning units were then identified independently and coded, representing different aspects of the participants’ experiences of threats or violence. The contents of each coded group were condensed, and then summarized to make generalized descriptions of how the threats and violence were dealt with. The final description was illustrated by selected quotations. All quoted participants were given pseudonyms.

All the authors are health care workers and researchers. One of the authors (IHJ) is a GP with clinical experience from EPC. The study was approved by the Regional Committee for Medical Research Ethics.

## Results

Threatening situations were familiar to all the participants, but not all participants had been physically hurt. Four main themes emerged regarding how the system influenced the incidents of threats or violence (Table 2): (1) minimizing the risk of working alone, (2) being prepared, (3) resolving the mismatch between patient expectations and the service offered, and (4) supportive manager response. The four themes are further elaborated below.

**Table 2:** **Emergency primary health care personnel’s dealing with violence from patients: MAIN themes and sub-themes**

**Main themes**	**Sub-themes**
Minimizing the risk of working alone	
	Ability to summon someone
	Colleagues turning up
Being prepared	
	Precautions when facing warning signs
	Education and training
Resolving the mismatch between patient expectations and service offered	
	Clarifying the role
	Improving the routines
Supportive manager response	

### Minimizing the risk of working alone

One of the main themes in all the focus group discussions was *minimizing the risk of working alone.* The experience of being alone in a threatening situation was commonplace. However, the narratives included experiences ranging from working completely alone without any colleagues on site, to situations in which colleagues or potential helpers had been present in the building, but out of sight or earshot. The discussions pointed to two factors which influenced the experience of being alone: the ability to summon someone, and chance intervention by colleagues.

Many of the participants emphasized the importance of having an alarm for the *ability to summon someone* when in a threatening situation. They claimed the available alarm system gave a sense of security, although it was seldom in use. However, just having an alarm system was not sufficient to feel safe. The efficiency of the alarm seemed to depend on the response time after the security assistant was alerted. If there was a long response time, or there was uncertainty about the responders’ alertness, the benefit of the alarm was diminished.

*…indeed, there is a red key that we can press (at the National Radio System). But really, I do not know what kind of security this alarm gives. Because where I am, it would take 10 to 15 minutes at a minimum before (help arrives). They (the visitors) would be able to do a lot of harm within that time span. (Gina, nurse)*

Another common experience of resolving threatening situations was *colleagues turning up* more or less by chance. Several participants related escalating conflicts which came to an end because someone appeared by chance. On the one hand, “by chance” was described as someone turning up and interrupting completely unexpectedly. On the other hand the “by chance” was described as colleagues checking on the situation after observing warning signs. A physician told about one episode she experienced whilst pregnant. A drugged male patient broke the window into the EPC centre and was soiled with blood. Alone with the physician in the consultation room, the patient threatened to kick her pregnant abdomen. She had no alarm or means to escape. She explained:

*Suddenly people just appeared. In that respect, it was by chance. But I rather think that they at least had a notion that we should watch out for each other, because of how the situation was when he (the patient) arrived.* (Milly, physician)

The narratives of situations being resolved by summoning colleagues, or by colleagues appearing by chance, were contrasted with stories where expected help was not received. One of the participants had experienced a situation in which she was threatened, and her colleague had escaped into another room and shut the door behind her. She explained how this experience increased her feeling of being left on her own.

### Being prepared

Some stories pointed to how the participants *prepared* for the possibility of a threatening or violent situation*.* One element in being prepared was participants’ own *precautions when facing warning signs*, like increased general alertness, obtaining as much information as possible before an expected complicated consultation, alerting colleagues to keep an eye on the situation, and involving the police upfront. One example of this strategy was when a physician was driven to home visits in a taxi, and the driver was told to enter the house if the physician did not return by an agreed time. Another strategy was to call the police before the consultation when warning signs like aggression or substance abuse were recognized. A third strategy was to ensure that other colleagues were on site when threatening situations were expected. A GP explained how he tried to secure the more inexperienced physicians:

*I have often suggested to them that they should schedule the appointment with them (the patients) at the start of their duty, so that I can sit here (in my office) for a while – leaving the door open …* (Nic, physician)

Some participants focused on the importance of being mentally prepared for the challenging and unsecure situations which sometimes emerge when working in EPC. Some of the participants said that they were better prepared due to *education and training* in managing threats and violence. Some had attended courses led by experts on security, such as police officers, psychologists or psychiatrists. Examples of useful knowledge were to be aware of own body language, to know how to de-escalate a potentially threatening situation and how to escape if necessary. The individual competence of the health professional, based on education and experience, was described as crucial when meeting patients in crisis situations.

### Resolving the mismatch between patient expectations and the service offered

Across the groups, participants related episodes of threats and violence that they perceived to be results of a *mismatch between patient expectations and the service offered*. Existing organizational routines – or lack of clear routines – sometimes created conflicts between patients and health workers. A typical example was inconsistency in how demands were handled for classified drugs, i.e. benzodiazepines. Several participants described a decrease in conflicts when consistent rejection of requests was enforced, and that this decreased the number of requests over time. Similarly, open-handed prescribers increased the demands and the conflicts. Another example was patients expecting consultations for minor issues, like coughs, sore throat and earache. The participants explained that patients who attended EPC centers for such conditions might experience long waits due to higher priority for more severe illnesses or injuries. The experience of not being prioritized, combined with the long waiting time, could lead to frustration, and in some cases this frustration created aggressive encounters.

One strategy to resolve the mismatch between patient expectations and the service offered was to guide the patient in proper use of the health care service and thereby *clarify the role of the EPC.* In meeting patients with minor issues, this sometimes included informing the patient about the priority and natural course of their condition, and also advising them where they might obtain more appropriate help, for example at their regular GP’s surgery. Some participants also actively defused conflict situations by informing patients of the possibility of submitting a formal complaint to the board of health supervision.

Inconsistent practice among physicians at the EPC center arising from varying attitudes and opinions made it difficult to argue for specific practices. Several participants therefore discussed the need for more consistent rules within the EPC. Some said that the continual challenge of meeting patient requests was solved by *improving the routines* at the EPC centre in order to reduce the mismatch between expectations and services offered. One example of an action taken was making the guidelines for contacting the EPC centre easily accessible to the public, and thereby trying to make a norm for proper usage. Another example was making a notice in the patient’s electronic medical record about not handing out drugs to that patient. The notification ensured some degree of consistency from the EPC centre independent of who was the health worker on site.

*It has really worked purposefully … towards that, in general you don’t get any drugs at the EPC centre. … When there are problems connected with substance abuse, there often is a notification about those patients that drugs shall not be given out. So, they possibly contact the EPC centre less … because they know they will not get anything.* (Henry, physician)

### Supportive manager response

Across the groups, participants described the manager’s key role in providing support in following up episodes. Many participants said that the manager’s attitude was crucial in dealing with the aftermath. Contrasting stories were presented: Stories of how a violent episode was taken seriously and followed up by the manager and, by contrast, stories in which the manager had left the health workers to their own devices. Examples of supportive responses were managers who contacted the police or who gave advice on how to manage the incident. Some participants also said that they had regular meetings at the EPC center where the health workers discussed recent incidents and how they were coped with. Security measures might be implemented as a result of the discussions after incidents. Examples were improved or new alarm systems, installing unbreakable glass at the entrance, or installing glass partitions between the reception and the waiting room.

Other participants told about a lack of routines for follow-up after a violent incident. Typically, health workers had to sort things out on their own initiative, and their story could be met with silence or belittlement. They had to report the case to the police and thereafter appear in court by themselves without any support from the employer. A nurse said that the feeling of being let down when she needed support was overwhelming, and she also thought her experience had influenced her colleagues negatively.

*The employer was not there for me. It was so unpleasant that I will never report again, no matter what happens. This is also the attitude among my colleagues. We still fill in the violence reports, but nothing really happens. They (the forms) are placed at the bottom of a drawer.* (Ally, nurse)

## Discussion

The results of this study suggest that important organizational factors in dealing with workplace violence are minimizing the risk of working alone, preparing the personnel for violent situations, reducing the mismatch between patient expectations and services offered, and supportive manger response. The findings underscore that control strategies need to address the interacting system factors that contribute to workplace violence [28]. The significance of the focus on the system is also supported by an Italian study, which concluded that changes in work organization at different levels contributed to reducing violence against health care workers [32].

Working alone has previously been reported as a risk factor for workplace violence in health care [28,33]. Although working alone is not in itself against the law, several countries have legislations that require employers to think about and deal with any health and safety risks before people are allowed to work alone [25,34]. According to the Norwegian Working Environment Act, special risks associated with working alone should be assessed. Further, measures for preventing and reducing any risk of working alone ought to be implemented in order to meet the statutory requirements of a satisfactory working environment [25]. One strategy is to assess the risk on a case-by-case basis, and take precautions accordingly, by using a risk assessment tool for safer lone working among health professionals [35]. Working in EPC includes several risk factors addressed in this assessment tool, such as unknown patients, and inability to obtain patient information before meeting the patient [26].

Supportive colleagues on site seemed highly significant in dealing with threats and violence. However, the results show that availability of staff does not guarantee social support. Studies on the relationship between number of staff and violent incidents suggest that the type and the competence of additional staff are more important than staff numbers [30].

The availability of an alarm system was also presented as essential for the management of threats and violence. Installation and maintenance of an alarm on the work premises is among the recommended action points to ensure safety and security, especially when working alone [33,35]. However, the existence of alarms is no guarantee for the feeling of safety; and the results of this study specifically point to the importance of short response time to alarms. Routines for follow-up and maintenance are also needed. In Norway this is supported by the Working Environment Act, which requires employers to systematically follow up, correct and make improvements if there are deficiencies [36].

The importance of increasing preparedness through training in the management of violence is supported by several studies [32,37]. A previous Norwegian study showed that only 40% of the EPC centers had systematic training regarding violence against personnel [26]. Providing education and training is thus an obvious possibility for increased safety and security, and this could even be provided at a national level.

The findings of this study draw attention to how a mismatch between patient expectations and offered services can be a challenge that has to be resolved in order to manage and even prevent violence at the EPC centers. Clarification of the role of the EPC, and communication with the public about the role and content of the service, seem to be central elements. Other studies have also found that violent behaviour is triggered by unmet client requests both in mental health care [30,38] and in out-of-hours GP care [39]. Cutcliffe has previously described inconsistency in the application of rules as well as clients’ reactions to rules or regimes as significant antecedents to violent incidents in mental health care [30]. These phenomena are also likely to be relevant for other health care organizations, like EPC centers. Training to improve communication with emotional, worried or anxious patients has been suggested to reduce aggressive behaviour [39]. In our opinion, a fruitful communication also has to be based on a system with clear and consistent rules for the service offered.

The manager at the EPC centers should provide support when violent episodes occur, as this was presented as crucial in the follow-up of incidents. Previous studies have underlined that when staff feel supported through formal support systems at work, there is an effect on such diverse factors as the quality of care offered to patients [40], emotional well-being and somatic health of the victim [41] and the recovery of victims of workplace violence [42]. These formal support systems should include the provision of legal and administrative advice, and implementation of organizational measures to prevent future incidents [43,44].

### Study strength and limitations

In our opinion, an understanding of violence based on the experiences from real incidents is a step forward in determining some of the factors in managing violence in the EPC. We received a large number of personal accounts describing incidents of threats and violence. The stories accounted for so many aspects, as well as recurring themes, that we considered the material to be saturated. The diversity in professional, geographical and organizational experiences among the participants increases the generalizability of the findings. By keeping the discussions within specific occupational groups, we diminished possible struggles for status and power between participants. We believe the homogenous groups increased the scope for openness, but the approach could also have disguised overt differences between professions and how they relate to each other. Due to practical reasons, there was a disparity in the number of participants. Two groups consisted of only two participants, which clearly limited the total range of experiences in these groups. Still, these groups added valuable nuances to the themes discussed in the larger groups.

## Conclusion

Our study shows a potential for development of better strategies for protecting the EPC personnel at risk of workplace violence. Possible strategies at the EPC centers are minimizing the risk of working alone, preparing the personnel for dealing with violent incidents, resolving the mismatch between patient expectations and service offered, and providing support in the follow-up of incidents. However, further studies are needed to assess the effectiveness of these measures in preventing and dealing with workplace violence.

## Abbreviations

EPC: Emergency primary health care
GP: General practitioner
